# Challenges in defining surgical-site infections following hydrocephalus surgeries

**DOI:** 10.1017/ice.2023.110

**Published:** 2023-12

**Authors:** Caitlin Naureckas Li, Amanda Bonebrake, Sangeeta Schroeder, Sandi K. Lam, Jeffrey S. Raskin

**Affiliations:** 1 Division of Pediatric Infectious Diseases, Department of Pediatrics, Ann & Robert H. Lurie Children’s Hospital, Chicago, Illinois; 2 Northwestern University Feinberg School of Medicine, Chicago, Illinois; 3 Center for Quality and Safety, Ann & Robert H. Lurie Children’s Hospital, Chicago, Illinois; 4 Department of Pediatrics, Ann & Robert H. Lurie Children’s Hospital, Chicago, Illinois; 5 Division of Hospital-Based Medicine, Department of Pediatrics, Ann & Robert H. Lurie Children’s Hospital, Chicago, Illinois; 6 Division of Pediatric Neurosurgery, Department of Neurological Surgery, Ann & Robert H. Lurie Children’s Hospital, Chicago, Illinois

## Abstract

Multiple organizations track neurosurgical surgical-site infection (SSI) rates, but significant variation exists among reporting criteria. We report our center’s experience with the variation in cases captured by 2 major definitions. Standardization could support improvement activities and SSI reduction.

Diversion of cerebrospinal fluid for hydrocephalus is one of the most common pediatric neurosurgical procedures, with >$2 billion in hospital charges per year.^1^ Surgical-site infections (SSIs) following shunt and related surgeries are a significant source of morbidity, with reported national averages nearing 10%.^2^ Multiple national organizations track these SSIs, but case definitions differ significantly. Three major organizations catalog hydrocephalus SSIs: (1) the Centers for Disease Control and Prevention’s National Healthcare Safety Network (NHSN), a national healthcare-associated infection monitoring system^3^; (2) Solutions for Patient Safety (SPS), a large collaborative of over 145 children’s hospitals focused on reducing multiple categories of healthcare-associated conditions^4^; and (3) the Hydrocephalus Clinical Research Network–Quality group (HCRNq), a large neurosurgery collaborative cohort.^2^ For hydrocephalus surgeries, the NHSN and SPS case definitions are identical; however, the HCRNq case definition has several key differences.

Data obtained from tracking SSIs are used in multiple ways. Measuring SSIs allows institutions to understand internal quality data and to prioritize areas for improvement. Additionally, reporting of quality data allows institutional benchmarking and can affect hospital reimbursement. Conflicting definitions can confound the interpretation and applicability of the data.

Prior work demonstrated vast differences between reporting criteria for SSI rates in other procedures.^5^ To better understand the real-world differences of reporting systems for hydrocephalus management procedures, we retrospectively reviewed all SSIs following neurosurgical shunt and related procedures at our institution.

## Methods

This study was conducted at our 360-bed quaternary-care children’s hospital with 7 board-certified pediatric neurosurgeons. We reviewed reporting criteria for 3 organizations to which our institution reports neurosurgical SSIs: the NHSN, SPS, and the HCRNq. We subsequently assessed the SSIs identified by each criterion between November 2020 and August 2022. Cases were identified through HCRNq reporting logs maintained by the Division of Pediatric Neurosurgery and the NHSN and SPS reporting logs are maintained by the hospital infection prevention and control (IPC) team. This work was deemed exempt from approval by our local institutional review board.

Neurosurgery shunts are defined as surgical implants that move spinal fluid in one direction from the brain to the peritoneal cavity, heart atrium, or pleural space. Neurosurgery shunt-related procedures, included in NHSN and SPS criteria but not the HCRNq criteria, include external ventricular drain placement and endoscopic third ventriculostomies.

## Results

Several major differences exist between the NHSN/SPS and the HCRNq case definitions, including length of time from index case, definitions of infection, and included ages. Notably, the HCRNq definition includes only patients aged <18 years, although our hospital, like many peer institutions, treats many patients aged >18 years with sequelae of conditions that began in childhood. Other key differences include procedure types eligible for inclusion, amount of time after a surgery during which a procedure is eligible for inclusion, and classification of culture-negative abdominal pseudocysts (Table 1).

**Table 1.** Comparison of Reporting Criteria

Reporting Group	National Healthcare Safety Network (NHSN)/Solutions for Patient Safety (SPS)	Hydrocephalus Clinical Research Network–Quality (HCRNq)
Timing since index procedure	30 days for superficial incisional SSI and 90 days for deep-incisional and organ-space SSI	No restriction
Procedure types included	Designated CPT codes as defined by NHSN that include shunt insertions/ and revisions, external ventricular drain placement, and third ventriculostomies	CPT codes for shunt insertions and revisions, externalizations, shunt explantations
Brief description of criteria	Evidence of infection including signs such a purulent drainage, positive culture, or abscess^a^	5 designated criteria: - CSF culture positive - Blood culture positive (ventriculoatrial shunts only) - Wound infection (not including simple stitch abscess) - Abdominal pseudocyst regardless of culture results - Exposed hardware
Age restriction	<109 years, (cases >18 years excluded from public reporting for pediatric hospitals)	<18 years
Index procedure excluded if invasive manipulation between index procedure and diagnosis?	Yes	No
Are procedures not performed in the OR excluded?	Yes	No

Note. CPT, current procedural terminology; CSF, cerebrospinal fluid; OR, operating room.

a For additional details, see the full Solutions for Patient operational definition for surgical-site infections at https://www.solutionsforpatientsafety.org/wp-content/uploads/sps-operating-definitions_January-2022-1.pdf.

**Figure 1.**
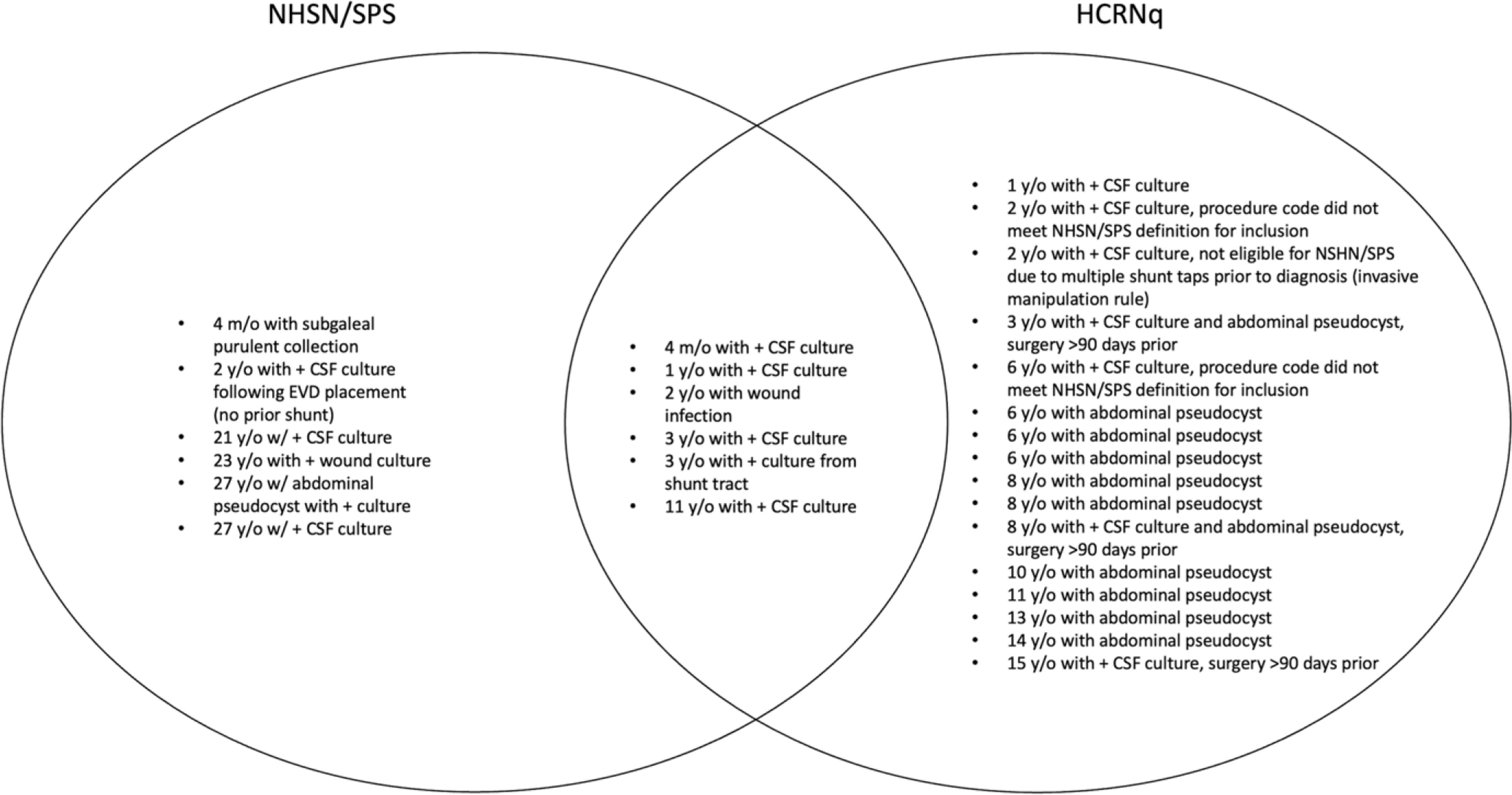
Brief case summaries of surgical site infections by reporting definition.

Between November 2020 and August 2022, a total of 265 neurosurgical shunt and related procedures were performed. Of those, 22 procedures (8.3%) had complications that met the HCRNq SSI definition, and 12 procedures (4.5%) met the SPS/NHSN definition. Only 6 procedures met criteria for reporting to both organizations (Fig. 1). Also, 4 patients had 2 infections each during the study period. All patients survived to the end of the study period.

## Discussion

Only limited overlap occurred among the cases that met each of the 2 major reporting definitions for hydrocephalus surgery SSIs. To our knowledge, this is the first report examining the real-world implications of these definitions.

An ideal SSI definition would capture all surgery-related infections regardless of age that were likely a result of the index procedure. HCRNq focuses exclusively on pediatric patients, and therefore limits reporting to patients aged <18 years; a 19-year-old patient with an SSI is not counted by HCRNq. Similarly, a patient returning with an SSI 91 days following the index procedure is not counted by the NHSN/SPS definition. Within our data set, many patients with clear SSIs would have been missed if only 1 of the existing definitions were applied. These examples underscore the limitations of these definitions in understanding an institutional infection rate.

Multiple adverse outcomes exist for which the definition of SSI is controversial. For example, it is generally accepted that the vast majority of shunt infections that occur as a direct result of the index procedure will manifest within the first 6 months.^6^ Therefore, whether infections occurring after the 6-month period should be counted in the same way as those that appear earlier remains an open question. Preoperative bundles and other similar preventative measures may not be the most effective strategies to address these infections. Additionally, the HCRNq criteria consider all abdominal pseudocysts to be SSIs regardless of whether an organism is cultured. Although some culture-negative pseudocysts may be the result of fastidious organisms that do not grow well in the laboratory, no consensus or strong data support the idea that they are all the result of an infectious organism. Therefore, similarly, it may be appropriate to track culture-negative pseudocysts separately from other SSIs because reducing their incidence may require different interventions.

Arguably the most important goal of SSI reporting is to allow institutions to understand their current state and to identify interventions to prevent future infections. Studies of the SPS network demonstrate that membership in the network results in an improvement in a variety of healthcare-associated conditions.^7,8^ Similarly, iterative protocolization through the HCRNq and its parent Hydrocephalus Clinical Research Network has shown that adherence to surgical implant bundles can drive down shunt infection rates.^2,9^ However, because each organization tracks infection rates using their own definitions, the impact on infections outside the scope of their definition is unclear. Arguably, an ideal reporting system would capture all surgeries with preventable infectious complications, maximizing the opportunities for organizations to recognize areas for intervention to prevent patient harm. Based on our findings, opportunities for standardization include removal of age restrictions for inclusion, consensus on the significance of abdominal pseudocysts, and agreement about the period after the procedure in which positive cultures suggest an SSI.

From a practical standpoint, the differences in reporting criteria place an additional administrative burden on hospitals. Administrative data alone have limited utility for tracking SSIs,^10^ so although some pieces of surveillance can be automated, many steps must be completed manually, often by an employee with advanced training such as a certified infection preventionist. If reporting criteria were standardized, redundant and time-consuming chart reviews could be streamlined.

This study had several limitations. The number of SSIs during the study period was small. Our hospital has only been reporting to HCRNq since late 2020; therefore, we could not include earlier cases. Additionally, our single-center design may limit the generalizability of our findings. However, the differences in reporting requirements apply to all participating centers. Our surgical case-mix index is similar to that of other centers contributing data to these organizations.

Future work in our hospital will include close collaborations between the IPC team, the Center for Quality and Safety, and quality improvement leadership within the Division of Pediatric Neurosurgery to ensure that all opportunities for improvement in SSI rates are considered. Other reporting will also be examined, such as the sampling methodology of the National Safety and Quality Improvement Project. Comprehensive and cohesive SSI reporting systems would be a vital tool in supporting future improvement work.
